# Global adoption of robotic technology into neurosurgical practice and research

**DOI:** 10.1007/s10143-020-01445-6

**Published:** 2020-11-30

**Authors:** Vittorio Stumpo, Victor E. Staartjes, Anita M. Klukowska, Aida Kafai Golahmadi, Pravesh S. Gadjradj, Marc L. Schröder, Anand Veeravagu, Martin N. Stienen, Carlo Serra, Luca Regli

**Affiliations:** 1 Machine Intelligence in Clinical Neuroscience (MICN) Lab, Department of Neurosurgery, Clinical Neuroscience Center, University Hospital Zurich, University of Zurich, Frauenklinikstrasse 10, 8091 Zurich, Switzerland; 2 School of Medicine, Università Cattolica del Sacro Cuore, Rome, Italy; 3 Amsterdam UMC, Neurosurgery, Amsterdam Movement Sciences, Vrije Universiteit Amsterdam, Amsterdam, Netherlands; 4 School of Medicine, University of Nottingham, Nottingham, UK; 5 HARMS (Human-centered Automation, Robotics and Monitoring for Surgery) Laboratory, Faculty of Medicine, Department of Surgery & Cancer, Imperial College London, London, UK; 6 Department of Neurosurgery, Leiden University Medical Centre, Leiden, The Netherlands; 7 Department of Neurosurgery, Erasmus MC, University Medical Centre, Rotterdam, The Netherlands; 8 Department of Neurosurgery, Bergman Clinics, Amsterdam, The Netherlands; 9 Neurosurgery AI Lab, Department of Neurosurgery, Stanford University, Stanford, CA USA

**Keywords:** Robotics, Robotic guidance, Technology, Neurosurgery, Global, Worldwide survey

## Abstract

Recent technological advancements have led to the development and implementation of robotic surgery in several specialties, including neurosurgery. Our aim was to carry out a worldwide survey among neurosurgeons to assess the adoption of and attitude toward robotic technology in the neurosurgical operating room and to identify factors associated with use of robotic technology. The online survey was made up of nine or ten compulsory questions and was distributed via the European Association of the Neurosurgical Societies (EANS) and the Congress of Neurological Surgeons (CNS) in February and March 2018. From a total of 7280 neurosurgeons who were sent the survey, we received 406 answers, corresponding to a response rate of 5.6%, mostly from Europe and North America. Overall, 197 neurosurgeons (48.5%) reported having used robotic technology in clinical practice. The highest rates of adoption of robotics were observed for Europe (54%) and North America (51%). Apart from geographical region, only age under 30, female gender, and absence of a non-academic setting were significantly associated with clinical use of robotics. The Mazor family (32%) and ROSA (26%) robots were most commonly reported among robot users. Our study provides a worldwide overview of neurosurgical adoption of robotic technology. Almost half of the surveyed neurosurgeons reported having clinical experience with at least one robotic system. Ongoing and future trials should aim to clarify superiority or non-inferiority of neurosurgical robotic applications and balance these potential benefits with considerations on acquisition and maintenance costs.

## Introduction

Neurosurgery is one of the most complex and delicate surgical specialties because of the limited maneuverability determined by the small surgical fields of modern minimally invasive approaches. Furthermore, high-precision standards are required to obtain maximal therapeutic benefits without compromising the function of noble anatomical structures of the central and peripheral nervous system [1]. Recent technological advancements have led to the development and implementation of robotic surgery in several specialties including general surgery, urology, gynecology, endocrine surgery, and orthopedics [2]. In this regard, neurosurgery—despite lagging behind the other specialties in terms of robotic applications because of its very technical peculiarities—constitutes no exception [1], and the practical application of robotic surgery is increasingly reported in the medical literature for the treatment of adult cranial [3], spinal [3–5], and pediatric pathologies [6].

Another reason for the rising importance of robotic technology in surgery is the advent of artificial intelligence in medicine. These advances have paved the way for the development of concepts such as the smart operating room, a futuristic surgical theater where human intervention is minimal, information is processed by smart objects, and decisions are made in an automated way. In such a setting, robots will have a major role not only in carrying out the surgical steps according to protocol but also as an intrinsically intelligent mind which can assess the environment and adjust accordingly in real time, or take appropriate actions to prevent errors [7, 8].

Even robotic technologies that have been widely applied in other specialties have often demonstrated less than satisfying clinical performance. In light of the increasing appeal that robotics is gaining in the neurosurgical field, its application in routine clinical practice needs to be solidly grounded on evidence, with proof of superiority or non-inferiority compared with traditional neurosurgical interventions [9]. Moreover, in addition to considerations of technical feasibility and possible impact on outcome improvement, the implementation of robotic technology has to take into account also the financial repercussions on the healthcare system inherent to the high acquisition and maintenance costs [10].

While other surveys have tried to describe the status of worldwide applications of new neurosurgical technologies like neuronavigation [11], and despite the encouraging apparent trend in increased applications of neurosurgical robotics with the resulting possible clinical benefit and research advancement, global data on the adoption of robotics in neurosurgical practice and research is currently lacking.

Our aim was to carry out a worldwide survey among neurosurgeons to assess the adoption of and attitude toward robotic technology in the neurosurgical operating room, and to identify factors associated with use of robotic technology.

## Materials and methods

### Sample population

The survey was distributed via the European Association of the Neurosurgical Societies (EANS) and Congress of Neurological Surgeons (CNS) in January, February, and March 2019. The EANS is the professional organization that represents European neurosurgeons. An e-mail invitation was sent through the EANS newsletter on January 28, 2019. Furthermore, the membership database of the CNS was searched for e-mail addresses of active members and congress attendants. The CNS is a professional, United States-based (US) organization that represents neurosurgeons worldwide. At the time of the search, the database contained 9007 members from all continents, a subset of which had functioning e-mail addresses. The survey was hosted by SurveyMonkey (San Matea, CA (USA)) and sent by e-mail together with an invitation letter. Reminders were sent after 2 and 4 weeks to non-responders to increase the response rate. To limit answers to unique site visitors, each e-mail address was only allowed to fill in the survey once. All answers were captured anonymously. No incentives were provided.

### Survey content

The online survey was made up of nine or ten compulsory questions, depending on the participants’ choice of whether they had or had not used robotic technology in their neurosurgical practice. A complete overview of survey questions and response options is provided in Table 1. The order in which potential reasons for use/non-use are displayed was randomized to avoid systematic bias. The definition of robotic technologies that was provided within the survey was: “Any form of robotic assistance in neurosurgery, including but not limited to cooperative robot arms and modules (“cobots“) assisting in surgical maneuvers such as pedicle screw placement, endoscopy, radiosurgery, microscopy, biopsy, or DBS electrode placement, etc.” The survey was developed by the authors based on prior, similar surveys carried out in a similar population. This report was constructed according to the Checklist for Reporting Results of Internet E-Surveys (CHERRIES) guidelines [12].

**Table 1:** Elements contained within the survey. Depending on the participants’ choice, nine or ten questions were displayed

Question	Response options	Type
What is your primary subspecialty?	Spine; neurovascular, neurooncology, trauma, epilepsy, pediatric, peripheral nerve, neurointensive care, functional; other	Single choice; free text
What setting do you primarily practice in?	Academic hospital, non-academic hospital, private practice, other	Single choice; free text
What is your level of experience?	Medical student, resident, fellow, board-certified/attending, chairperson, other	Single choice; free text
What is your gender?	Male, female	Single choice
What age group are you in?	< 30 years, 30–40 years, 40–50 years, 50–60 years, > 60 years	Single choice
What country are you currently based in?	List	Single choice
In your clinical practice, have you ever made use of robotic technology?	Yes, No	Single choice
If yes		
Which robotic device(s) do you use/have you used?	–	Free text
Please rate the importance of the following reasons for using robotic assistance from 1 to 4, based on your own clinical experience		
Improved cost-effectiveness	1 (Not important) to 4 (Highly important)	Single choice
Time savings	1 (Not important) to 4 (Highly important)	Single choice
Improved surgical outcome	1 (Not important) to 4 (Highly important)	Single choice
Lower risk of complications	1 (Not important) to 4 (Highly important)	Single choice
Attract patients and referrals/marketing	1 (Not important) to 4 (Highly important)	Single choice
If no		
Please rate the importance of the following reasons for not using robotic assistance from 1 to 4		
Lack of published supporting evidence	1 (Not important) to 4 (Highly important)	Single choice
Acquisition/maintenance costs	1 (Not important) to 4 (Highly important)	Single choice
Difficulties with staff training/device education	1 (Not important) to 4 (Highly important)	Single choice
Not personally convinced by their added value	1 (Not important) to 4 (Highly important)	Single choice
No demand for robotic assistance/lack of applicable devices	1 (Not important) to 4 (Highly important)	Single choice
In your research, have you ever made use of robotic technology?	Yes, No, I do not engage in medical research	Single choice

### Statistical analysis

Continuous variables are given as means ± standard deviations (SD), whereas categorical variables are reported as numbers (percentages). Countries were grouped by region (Europe/North America/Latin America/Asia & Pacific/Middle East/Africa) according to a previous worldwide survey by Härtl et al. [11]. Fisher’s exact test was applied to compare implementation incidence of robotics among regions. By use of a multivariate logistic regression model, we identified independent predictors of adoption of robotic technology into clinical practice and research, respectively. The importance of reasons for use or non-use of robotics was compared among regions using the Kruskal-Wallis *H* tests. When calculating the ratio of respondents who had applied robotic technology in research, we incorporated both respondents who had never used robotics in their research and those who do not participate in medical research into the denominator. R version 3.5.2 (The R Foundation for Statistical Computing, Vienna, Austria) was applied for all analyses, and the Type I error rate was defined as *p* ≤ 0.05 for two-tailed tests.

## Results

### Response rate and respondent characteristics

From a total of 7280 neurosurgeons who were sent the survey, we received 406 answers, corresponding to a response rate of 5.6%. Detailed characteristics of the respondents are given in Table 2. The majority of respondents were in the 30–40 years age group (33%), and 88.7% of the answers were from male participants. Most of surveyed neurosurgeons were specialized in spinal surgery (34.5%). As far as the work setting was concerned, more than two-thirds of the neurosurgeons were practicing in an academic hospital (67.7%), followed by non-academic hospital (15.5%), private practice (15%), and other settings (1.7%). We also sought to describe the level of experience of the surveyed population. Participants were mostly board-certified/attending neurosurgeons (58.9%), while residents (20%), chairs of department (10.8%), fellows (4.7%), medical students (3.2%), and others (2.5%) were less represented. Geographic distribution of the answers was skewed in favor of North America (70.4%) and Europe (17.2%), while less answers were received from surgeons from Asia and Pacific (5.4%), Latin America (3.9%), Middle East (2.5%), and Africa (0.5%).

**Table 2:** Basic demographics of the surveyed population

Parameter	Value (*n* = 406)
Age group (years), *n* (%)	
< 30	38 (9.4%)
30–40	134 (33.0%)
40–50	102 (25.1%)
50–60	66 (16.3%)
> 60	66 (16.3%)
Male gender, *n* (%)	360 (88.7%)
Subspecialty, *n* (%)	
Spine	140 (34.5%)
Neuro-oncology	74 (18.2%)
Neurovascular	56 (13.8%)
Pediatric	38 (9.4%)
Functional	36 (8.9%)
Trauma	31 (7.6%)
Epilepsy	19 (4.7%)
Neurointensive care	4 (1.0%)
Skull base	5 (1.2%)
Peripheral nerve	2 (0.5%)
Other	1 (0.2%)
Work setting, *n* (%)	
Academic hospital	275 (67.7%)
Non-academic hospital	63 (15.5%)
Private practice	61 (15.0%)
Other	7 (1.7%)
Level of experience, *n* (%)	
Board-certified/attending	239 (58.9%)
Resident	81 (20.0%)
Chairperson	44 (10.8%)
Fellow	19 (4.7%)
Medical student	13 (3.2%)
Other	10 (2.5%)
Region, *n* (%)	
North America	286 (70.4%)
Europe	70 (17.2%)
Asia Pacific	22 (5.4%)
Latin America	16 (3.9%)
Middle East	10 (2.5%)
Africa	2 (0.5%)
Use of robotic technology in clinical practice, *n* (%)	197 (48.5%)
Use of robotic technology in clinical research, *n* (%)	209 (61.5%)

### Robotics in clinical practice and research

**Fig. 1:**
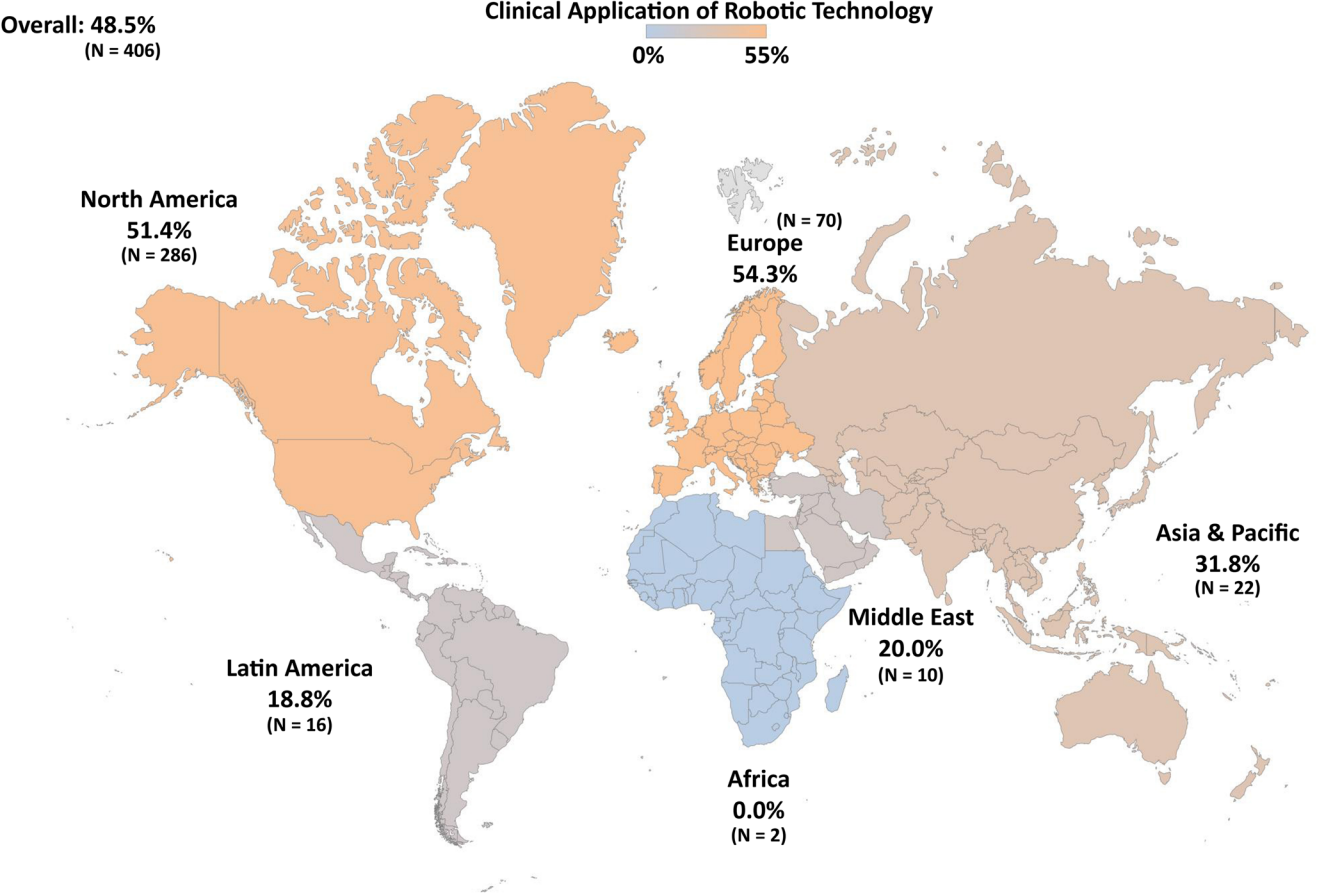
Proportions of neurosurgeons who report having used robotic technology in their clinical practice among the 406 responders, stratified by region and plotted on a world map (Mercator projection)

When inquired about the use of robots in neurosurgical clinical practice and research, 48.5% and 61.5% of the surveyed population answered positively, respectively. Stratified by region (Table 3), use of robotic technology in clinical practice was most common in Europe (54.3%) and North America (51.4%), followed by Asia and Pacific (31.8%), Middle East (20.0%), Latin America (18.8%), and Africa (0.0%). Figure 1 provides a graphical illustration of the worldwide clinical use of robotics in neurosurgery. Respondents were also asked to list which types of robots they had worked with (Table 4). The most commonly used robotic devices were from the Mazor family (32%), followed by the ROSA robot (26.4%). A high proportion of the robot users did not identify the specific type of robots that they had used (33.5%).

**Table 3:** Application of robotic technology in clinical practice and research, stratified by region

Domain	Region							*p*
	Overall (*n* = 406)	North America (*n* = 286)	Europe (*n* = 70)	Latin America (*n* = 16)	Asia Pacific (*n* = 22)	Middle East (*n* = 10)	Africa (*n* = 2)	
Clinical practice, *n* (%)	197 (48.5)	147 (51.4)	38 (54.3)	3 (18.8)	7 (31.8)	2 (20.0)	0 (0.0)	0.008*
Clinical research, *n* (%)^a^	85/369 (20.9)	50/255 (19.6)	26/68 (38.2)	2/15 (13.3)	5/20 (25.0)	1/9 (11.1)	1/2 (50.0)	0.021*

*p ≤ 0.05

^a^While all responders answered the question on robotic use in clinical practice, a subset did not answer the second question on application of robotic technology in clinical research

**Table 4:** Most commonly reported robotic devices

Device	Value (*n* = 197)
Mazor Family, *n* (%)	
Overall	63 (32.0%)
Undefined	50 (25.4%)
SpineAssist	6 (3.0%)
Renaissance	5 (2.5%)
Mazor X	2 (1.0%)
ROSA, *n* (%)	52 (26.4%)
Excelsius GPS, *n* (%)	12 (6.1%)
Neuromate, *n* (%)	10 (5.1%)
Cirq, *n* (%)	9 (4.6%)
DaVinci, *n* (%)	7 (3.6%)
Synaptive, *n* (%)	5 (2.5%)
Cyberknife, *n* (%)	4 (2.0%)
Visualase, *n* (%)	2 (1%)
Corindus, *n* (%)	1 (0.5%)
Others/unspecific, *n* (%)	66 (33.5%)

#### Predictors of robotics use

Multivariate logistic regression analysis was used to investigate independent predictors of adoption of robotics into clinical practice and research (Table 5). Tested variables included age, gender, specialty, work setting, surgeon experience, and geographic region of origin. The analysis revealed that after adjustment for potential confounders, young surgeons (< 30 years) were more likely than those belonging to other age ranges to have used robotic technology in clinical practice (OR 2.55, CI 1.26–5.23, *p* = 0.010). Other relevant results include the lower likelihood of male (OR 0.46, CI 0.21 to 0.96, *p* = 0.042) and non-academic neurosurgeons (OR 0.45, CI 0.23–0.87, *p* = 0.019) to have clinically used robotic technology in neurosurgery. Also, surveyed surgeons from Asia Pacific (OR 0.15, CI 0.03–0.54, *p* = 0.008) and Middle East (OR 0.14, CI 0.02–0.57, *p* = 0.028) were significantly less likely to implement robotics application in clinical practice compared with North America as the reference category. The only independent predictor of use of robotic technology in clinical research was a European region of origin (OR 2.15, CI 1.1–0.4.21, *p* = 0.025).

**Table 5:** Multivariate logistic regression analysis for characteristics associated with relationship between adoption of robotics into clinical practice and research, respectively

Parameter	Clinical practice			Clinical research		
	OR	95% CI	*p*	OR	95% CI	*p*
Age group						
< 30	2.55	1.26 to 5.23	0.010*	1.46	0.59 to 3.54	0.401
30–40	Reference	–	–	Reference	–	–
40–50	1.68	0.84 to 3.40	0.142	2.14	0.92 to 3.03	0.078
50–60	1.61	0.78 to 3.35	0.197	1.16	0.43 to 2.96	0.766
> 60	1.35	0.41 to 4.35	0.619	1.50	0.35 to 6.14	0.574
Male gender	0.46	0.21 to 0.96	0.042*	1.55	0.65 to 4.06	0.347
Subspecialty						
Spine	Reference	–	–	Reference	–	–
Neuro-oncology	1.37	0.70 to 2.71	0.352	0.71	0.32 to 1.55	0.396
Neurovascular	0.63	0.31 to 1.26	0.196	0.74	0.32 to 1.63	0.461
Pediatric	0.75	0.32 to 1.71	0.495	0.39	0.11 to 1.1	0.093
Functional	1.38	0.61 to 3.19	0.444	0.51	0.16 to 1.43	0.229
Trauma	0.90	0.38 to 2.14	0.806	0.58	0.19 to 1.55	0.301
Epilepsy	0.47	0.15 to 1.35	0.170	0.40	0.08 to 1.47	0.206
Neurointensive care	NA	NA	0.983	NA	NA	0.986
Peripheral nerve	0.85	0.03 to 23.5	0.915	NA	NA	0.853
Skull base	NA	NA	0.076	1.25	0.06 to 11.44	0.988
Other	NA	NA	0.991	NA	NA	0.991
Setting						
Academic	Reference	–	–	Reference	–	.
Non-academic	0.45	0.23 to 0.87	0.019*	0.44	0.17 to 1.04	0.073
Private practice	0.57	0.29 to 1.11	0.103	0.70	0.30 to 1.55	0.392
Other	0.84	0.15 to 4.32	0.832	0.82	0.04 to 6.56	0.867
Experience						
Board certified/attending	Reference	–	–	Reference	–	–
Resident	0.66	0.29 to 1.5	0.328	1.28	0.48 to 3.41	0.622
Chairperson	1.37	0.62 to 3.02	0.432	0.98	0.37 to 2.43	0.972
Fellow	4.85	1.13 to 3.43	0.057	1.72	0.44 to 6.3	0.421
Medical student	1.08	0.24 to 5.31	0.919	3.23	0.51 to 2.16	0.215
Other	0.61	0.12 to 2.56	0.501	2.16	0.41 to 9.41	0.322
Region						
North America	Reference	–	–	Reference	–	–
Europe	1.23	0.67 to 2.26	0.495	2.15	1.1 to 4.21	0.025*
Latin America	0.63	0.21 to 1.76	0.390	0.58	0.09 to 2.34	0.496
Asia Pacific	0.15	0.03 to 0.54	0.008*	2.06	0.58 to 6.5	0.232
Middle East	0.14	0.02 to 0.67	0.028*	0.41	0.02 to 2.8	0.444
Africa	NA	NA	0.987	NA	NA	0.220

*OR* odds ratio, *CI* confidence interval

*p ≤ 0.05

#### Attitudes toward robotic technology in neurosurgery

The surveyed population was asked to rate the importance of the factors for and against the use of robotic technology in neurosurgical clinical practice (Table 6). Among those surgeons implementing the use of robotic technology, the perceived improved surgical outcome (3.3 ± 0.9) and marketing considerations for augmentation of patient referrals (3.2 ± 0.9) were rated the most important, followed by time savings (2.7 ± 1.0), lower risk of complications (2.7 ± 1.0), and cost-effectiveness (2.3 ± 1.0). Only for time savings, we identified a significant difference in importance rating among the five regions (Kruskal-Wallis test, *p* = 0.003)—time savings were rated highly important in the Middle East and in Asia and Pacific, while this potential advantage was only of minor importance in Latin America.

**Table 6:** Tabulation of reasons for use and nonuse, per region. Responders graded importance of these reasons from 1 (not important) to 4 (highly important)

Parameter	Region							*p*
	Overall	North America	Europe	Latin America	Asia Pacific	Middle East	Africa	
Reasons for use								
Improved cost effectiveness	2.3 ± 1.0	2.4 ± 0.9	2.1 ± 1.2	1.7 ± 0.8	3.0 ± 0.0	3.0 ± 1.4	NA	0.072
Time savings	2.7 ± 1.0	2.9 ± 0.9	2.4 ± 1.1	1.7 ± 0.5	3.5 ± 0.7	3.0 ± 1.4	NA	0.003*
Improved surgical outcome	3.3 ± 0.9	3.4 ± 0.9	2.9 ± 1.1	2.9 ± 1.2	3.5 ± 0.7	4.0 ± 0.0	NA	0.057
Lower risk of complications	2.7 ± 1.0	3.2 ± 0–9	3.1 ± 1.0	2.6 ± 1.3	3.5 ± 0.7	3.5 ± 0.7	NA	0.648
Attract patients and referrals/marketing	3.2 ± 0.9	2.7 ± 1.0	2.8 ± 1.1	3.0 ± 0.6	3.0 ± 0.0	2.5 ± 2.1	NA	0.869
Reasons for non-use								
Lack of published supporting evidence	2.4 ± 1.0	2.4 ± 1.0	2.0 ± 0.9	2.9 ± 1.1	2.6 ± 1.0	2.6 ± 0.8	1.5 ± 0.7	0.061
Acquisition/maintenance costs	3.4 ± 0.9	3.4 ± 0.9	3.1 ± 1.0	3.3 ± 1.2	3.7 ± 0.6	3.9 ± 0.4	4.0 ± 0.0	0.054
Difficulties with staff training/device education	2.3 ± 1.0	2.4 ± 1.0	1.8 ± 0.8	2.7 ± 1.0	2.5 ± 1.0	2.4 ± 1.0	3.0 ± 1.4	0.030*
Not personally convinced by their added value	2.4 ± 1.1	2.6 ± 1.1	2.0 ± 1.1	2.0 ± 1.0	2.0 ± 1.0	1.9 ± 0.7	1.0 ± 0.0	0.008*
No demand for robotic assistance/lack of applicable devices	2.6 ± 1.0	2.6 ± 1.0	2.6 ± 1.0	2.6 ± 1.2	2.5 ± 0.8	2.7 ± 1.0	1.0 ± 0.0	0.424

Importance is presented as mean ± SD. The importance of reasons for use or non-use of robotics was compared among regions using the Kruskal-Wallis *H* tests

*p ≤ 0.05

Among those neurosurgeons who had never used robotics in clinical practice, the most important factor prohibiting adoption of robotics into clinical practice was the inherent acquisition/maintenance costs (3.4 ± 0.9). Other considerations played a lesser role in this choice. Of note, a statistically significant imbalance was found among regions with respect to difficulties with staff training and device education and also of personal convincement of the added value granted by the implementation of robotics in surgical practice (Kruskal-Wallis test, *p* = 0.030 and *p* = 0.008 respectively).

## Discussion

Our survey addressed a geographically diverse cohort of neurosurgeons at different levels of training. It is apparent that robotic surgery seems to have gained wide acceptance in neurosurgical practice as confirmed by the observation that almost half of the surveyed population have used robotic technology during neurosurgical procedures. Furthermore, around one-fifth of the surveyed population appears to have engaged in medical research using robotic technology. The overwhelming majority of robotics users was to be found in individuals under 40 years of age. Spinal surgery was the subspecialty that most often applied robotics, followed by neuro-oncologists, and cerebrovascular specialists. The most commonly used devices were the Mazor family and ROSA robots.

The proportion of neurosurgeons who reported having used robotic technology in clinical practice was very high and certainly higher than expected. Although, with recent trends, these numbers are conceivable, there are some factors that may potentially have led to a higher proportion of neurosurgeons reporting use of robotics in the surveyed population. First, the survey was circulated among EANS/CNS members and congress attendants, by way of which a potentially more scientifically interested and academic population was selected for. As observed in our survey, academic neurosurgeons are far more likely to have had contact with robotic surgery than their non-academic counterparts are. Second, it is possible and conceivable that among the population that was sent this survey, the surgeons with prior experience with robotics were more interested in this topic and therefore more likely to fill in a survey on robotic surgery (response bias). Even though these potential biases may have increased the proportion of neurosurgeons reporting clinical use of robotic technology, our results demonstrate that in recent years, robotics has seen broad adoption into the neurosurgical operating rooms of particularly Europe and North America.

After adjustment for potential confounders, no subspecialty was found to be significantly associated with an increased or decreased robotics use, neither in clinical practice nor in research. This suggests that robotic technology has been rather broadly applied in many neurosurgical subspecialties and for the treatment of several different pathologies. The main reasons guiding the increased implementation into clinical practice were the perceived improved surgical outcome granted by robotics as well as marketing considerations, potentially leading to more patient referrals. Predictably, adoption of robotic surgery into clinical practice was more frequent among younger surgeons, particularly those under 30, and less common in physicians practicing in non-academic centers. The fact that the use of robots in neurosurgery was particularly frequent in those < 30 years of age shows that neurosurgeons have increasingly contact with robotic technology during their residency training. The lower odds ratio identified for male respondents, may reflect an increased representation of the female population among the younger neurosurgeons and an encouraging trend in terms of closing the existing gender gap in neurosurgery [13, 14].

A statistically significantly decreased application of robotic surgery into clinical practice was found in Asia and Pacific and the Middle East compared with Europe and North America. In addition, lower clinical adoption was observed in Latin America and Africa, but this effect was not statistically significant due to the low sample size. These findings are compatible to the potentially decreased availability of resources in some of the countries belonging to the aforementioned regions. This hypothesis is also confirmed by a trend toward higher scores obtained for acquisition and maintenance costs as a reason for non-use of robotics with respect to other countries.

### Robotics in neurosurgery

The very definition of robotics poses some difficulties in identifying how neurosurgery is adapting to this increasingly evolving field. To date, most surgical robotics are very limited in their ability to perform procedures and make decisions automatically without major human intervention. Therefore, several other classifications have been proposed to describe surgical robots, based on one side on the device’s function and application, and on the other on the surgeon-robot interaction [15]. In fact, robotics far from only substituting and transforming the surgical act of the physician through automation and remote control has also been increasingly adopted for assisting specific surgical tasks, for example, anatomical localization of the lesion, stabilization of the surgeon’s hand during prolonged microsurgical work, or pedicle screw insertion [16, 17]. Moreover, the inherent complexity of neurosurgical procedures often requires different robotic competencies in different phases of surgery [1]. This kind of robotic aid is more precisely referred to as “cobot surgery”, where robotics enhance and maximize specific parts of the surgical procedure without performing automatic actions. Regardless, the use of robotic systems has been increasingly often reported in the neurosurgical literature, both for cranial and spinal applications [16, 18]. Table 7 provides an overview of relevant publications on the most recent developments of robotics in the field of neurosurgery.

**Table 7:** Recent narrative and systematic reviews on robotics in neurosurgery

Author	Year	Journal	Study design	N. studies	Collected data or investigated aspects	Robotic technology	Main findings
Marcus et al	2013	Eur Spine J	Systematic Review	5	Screw position accuracy (*n* = 5), LOS (*n* = 3), radiation exposure (*n* = 5)	SpineAssist (Mazor) VS fluoroscopy-guided surgery	Mixed results, insufficient reporting of study bias, surgeon proficiency in RA technology difficult to assess, different outcome measures, high costs. Future studies needed
Joseph et al	2017	Neurosurgical Focus	Systematic review	25	Accuracy of screw placement (*n* = 22), surgeon learning curve (*n* = 9), radiation exposure (*n* = 10), and reasons for robotic failure (*n* = 12)	Mazor (SpineAssist, Renaissance) ROSA	↑ surgical accuracy in RA instrumentation Radiation exposure unclear and dependent on technique and robot type
Menaker et al	2017	J NeuroIntervent Surg	Review	NA	Technologies under development for cerebrovascular and endovascular neurosurgery (RA-angiography, guided operative microscopes, coil insertion systems, endoscopic clipping devices)	Master-slave system for catheter guidance, robotic DSA system, mechanical coil insertion system, multisection continuum robot, auto-navigating microscope	Limits represented by logistical considerations, few experimental data, delays in emergency situations Many technologies under development but further studies needed Robotic systems in other interventional specialties have potential applications to endovascular neurosurgery but require modifications.
Ghasem et al	2018	Spine	Systematic review	32	Radiation exposure (*n* = 13), operative time (*n* = 13), accuracy (*n* = 15), length of stay (*n* = NA), complications/revision (*n* = NA)	Mazor (Renaissance, Mazor X), Rosa	Intrapedicular accuracy in screw placement and subsequent complications were = if not ↑ to the robotic surgery cohort Operative time ↑ in RA surgery compared to FH. Radiation exposure variable between studies; radiation time ↓ in robot arm as the number of robotic cases ascended (learning curve effect?) Multi-level procedures tend toward earlier discharge in patients undergoing robotic spine surgery
Fomenko et al	2018	Neurosurgery	Systematic review	35	Robotics in cranial neurosurgery (stereotactic biopsy, DBS and stereoelectroencephalography electrode placement, ventriculostomy, and ablation procedures)	PUMA, Minerva, Zeiss MKM. NeuroMaster, Neuromate, PathFinder, SurgiScope, ROSA, Renaissance, iSYS1	Cranial robotic stereotactic systems feature serial or parallel architectures with 4 to 7 degrees of freedom, and frame-based or frameless registration Indications for robotic assistance are diverse Low complication rates (++ hemorrhage)
Fiani et al	2020	Neurosurgical Review	Review	75	Accessibility (costs), health care quality (accuracy and precision, decrease in complication rate), cost-effectiveness (fluoroscopy time, OR time, revision rate)	Mazor’s SpineAssist/Renaissance	Accuracy, effectiveness, and safety of the RA surgery are convincing. Data on cost-effectiveness limited.
Molliqaj et al	2020	World Neurosurgery	Review	NA	Clinical outcome (pain, revisions, LOS, OR time, radiation); Radiological outcome (accuracy)	SpineAssist, Renaissance, Mazor X, ROSA, Excelsius GPS, TiRobot, DaVinci	Increased accuracy and safety in spinal instrumentation, reduction in surgical time and radiation exposure

*FH* free-hand, *LOS* length of stay, *NA* not available, *RA* robot-assisted

### Spinal applications

Several robotic systems are available for spinal interventions, mostly for assistance in pedicle screw placement [19]. Recent literature reported that robot-assisted screw placement is at least non-inferior if not superior with respect to accuracy than conventional free-hand technique and potentially decreases the rate of revision procedures [5, 17, 20–24]. A recent paper by Joseph et al. systematically reviewed applications of robotics in spinal surgery [18]. The authors reported that most comparative studies—apart from 1 RCT [25]—demonstrated that robotics can provide increased radiological accuracy with respect to free-hand placement both with the Mazor family and ROSA robots. A recent meta-analysis investigating clinically relevant pedicle screw revision in robotic-guided, navigated and freehand thoracolumbar instrumentations found that both robotics and navigation reduced post-operative revisions, but statistical significance was lost at sensitivity analysis for the former [9]. When length of hospital stay and overall complications were evaluated, Siccoli et al. showed that free-hand thoracolumbar screw insertion had worse results with respect to navigation, while no difference was found with robot-guided surgery [26]. On the contrary, no significant difference was found when radiation exposure was compared between robot-guided, navigated surgery, and free-hand approach [26]. More recently, a meta-analysis by Fatima et al. reported that perfect and acceptable pedicle screw accuracy as categorized by Gerztbein-Robbin classification was higher in robot-assisted than in free-hand surgery; complication rate, proximal facet joint violation, and intra-operative radiation time and exposure were significantly lower, while length of surgery was significantly higher [27]. Table 8 summarizes the results of most recent meta-analyses comparing robot-assisted spine surgery with navigated and free-hand technique.

**Table 8:** Recent systematic reviews and meta-analysis of robotics in spinal neurosurgery

Author	Year	Journal	N. studies	Intervention	N. patients	Outcome	Complications	Radiation exposure	Surgical time	Others
Staartjes et al	2018	World Neurosurgery	37	Thoracolumbar screw (FH vs NV vs RA)	7095	Screw revision: Intra-op—no difference Post-op—RA and NV ↓ than FH	–	–	–	–
Siccoli et al	2019	World Neurosurgery	32	Thoracolumbar screw placement (FH vs NV vs RA)	24,008	Accuracy No statistically significant differences among RG and FH (all *p* > 0.05). Lack of statistical power!!!	Compared with NV, FH ↑ overall complications (OR, 1.6; 95% CI, 1.3–1.9; *p* < 0.001).	Both RG and NV: no ↑ radiation use, compared with FH (both *p* > 0.05).	–	LOS (D, 0.7 days; 95% CI, 0.2–1.2; *p* = 0.006)
Perdomo-Pantoja et al	2019	World Neurosurgery	78	Screw placement (FH vs FA vs NV vs RA)	7858	RA and CTNav ↑ PS accuracy in thoracic spine than FH. NV—↑ PS placement accuracy than FA and RA (*p* < 0.01 and 0.04).	Patient revision rate FA ↑ than FH and NV (*p* < 0.01 and *p* < 0.01, respectively). Screw revision rate: FA ↑ than FH (*p* < 0.01)	–	–	Minor breach rate: NV ↓ than FH (*p* < 0.02), FA (*p* < 0.01), and RA (< 0.01). No differences among others (*p* > 0.059). Major breach rate: FH ↑ than NV (*p* < 0.04). No differences among the others (*p* > 0.05)
Fatima et al	2020	The Spine Journal	19	Screw placement (RA vs FH)	1525 (777 RA/ 748 FH)	Perfect placement: RA ↑ (OR 1.68, 95%CI 1.20–2.35, *p* = 0.003) Acceptable placement: RA ↑ (OR 1.54, 95%CI 1.01–2.37, *p* = 0.05)	Hardware failure, surgical revision, wound infections and neurological deficits. ↓69% in RA (OR 0.31, 95%CI 0.20–0.48, *p* < 0.00001)	↓ radiation time in RA (MD: − 5.30, 95%CI: − 6.83 to − 3.76, *p* < 0.00001) ↓ intra-op radiation doses in RA (MD: − 3.70, 95%CI: − 4.80 to − 2.60, *p* < 0.00001)	RA longer (MD 22.70, 95%CI 6.57–38.83, *p* = 0.006)	Proximal facet violation 92% ↓ in RA (OR 0.08, 95%CI 0.03–0.20, *p* < 0.00001)
Peng et al	2020	Annals of Translational Medicine	7 RCTs	Screw placement (RA vs FH)	540	Accuracy TiRobot-assisted technique ↑ SpineAssist-assisted technique ↓, Renaissance similar to conventional FH	–	RA ↓ (MD, − 12.36 s; 95% CI: − 17.92 to − 6.81 s; *p* < 0.0001)	RA ↑ (MD, 15.12 min; 95% CI 7.63–22.60 min; *p* < 0.0001)	–

*CI* confidence interval, *FA* fluoroscopy-assisted, *FH* free-hand, *NV* navigation, *PS* pedicle screw, *RA* robot-assisted, *RCT* randomized controlled trial, *WNS* World Neurosurgery

Highly powered ongoing prospective studies like the European Robotic Spinal Instrumentation (EUROSPIN) [12] and MIS-ReFRESH [7] studies are necessary to investigate if these potential benefits warrant the high acquisition and maintenance costs of these systems.

### Neuro-oncology

Robotic applications can also find applications in neuro-oncology. Most notably—of course also because invented by a neurosurgeon—the CyberKnife is one worldwide-adopted robot that is frequently used to treat tumors of all kinds using frameless stereotactic radiosurgery [28]. As other examples, robot-guided convection-enhanced delivery of chemotherapy for brainstem glioma was reported whereby the feasibility of accurately and safely delivering very small diameter catheters to deep targets within the brainstem was demonstrated [29]. Another example is the NeuRobot, a remotely controlled endoscope for tele-controlled tumor resection [30], which has been proven to be useful also for intraventricular dissections [31].

### Cerebrovascular/endovascular neurosurgery

Robotics is also gaining momentum in cerebrovascular and endovascular neurosurgery [32]. Currently tested applications (in vitro and in vivo) include cerebral angiography (also a robotic digital subtraction angiography (DSA) system), robot-assisted operating microscopes for the treatment of arteriovenous malformations and cavernomas, mechanical coil insertion systems for aneurysm treatment (reducing the number of operators needed for the procedure from two to one), and robotic endoscopic aneurysm clipping [33–35]. Moreover, several robotic systems that are already approved for clinical applications in other specialties like interventional cardiology and radiology may find fertile soil in neurosurgery after appropriate modifications [36].

### Other cranial applications

Other clinical applications of robotics systems in cranial neurosurgery include stereotactic biopsy targeting, deep brain stimulation (DBS) electrode placement, radiosurgery, placement of stereoelectroencephalographic (SEEG) electrodes for investigation of refractory epilepsy, ventricular catheter placement, and laser ablative procedures [16]. Growing interest is currently being placed on exoscopic camera systems to improve illumination and depth-of-field when difficult-to-access or deep lesions limit the visibility, although their potential advantages over traditional operating microscopes still remain questionable. For example, several small case series have addressed the efficacy and safety of the Synaptive Modus V exoscope system in both spinal and cranial surgery, with encouraging results [37].

### Limitations

Survey-based studies, while providing important insights, have inherent limits because of several potential biases. During survey distribution, selection and response bias are possible. Time constraints on responders may have limited their ability to answer with maximal accuracy, and in fact, concerning the adoption of robotic systems into clinical research, we obtained several incomplete or blank answers. The data is mostly based on subjective impressions of surgeons. Knowing this, bias could arise from the fact that surgeons who are more exposed to neurosurgical robotics can value it more positively than those who do not routinely make use of it, and vice-versa. However, reasons for advantages and disadvantages were specifically captured separately for users and non-users. Additionally, the relative percentage of geographic regions was skewed in favor of western countries, limiting the sensitivity of our survey for what concerns regions such as Asia and Pacific, South America, and in particular Africa.

## Conclusions

Our study provides a worldwide overview of neurosurgical adoption of robotic technology. Robotic systems have the technical potential to improve surgical procedures in terms of efficacy and safety by several means, spanning from indirect assistance of surgeons in complex parts of the operation (such as lesion localization) to more or less integral substitution of the manual skills required by the surgical task. Our survey sheds light on the diffusion of such technology and their general perception by neurosurgical specialists. Almost half of the surveyed neurosurgeons reported having clinical experience with at least one robotic system. The Mazor family and ROSA robots were most commonly applied. Before a consistent and widespread shift in clinical practice, superiority or non-inferiority of neurosurgical robotic applications needs to be established by high level of evidence studies and, at the same time, carefully balanced with considerations on costs of implementation. The results of ongoing and future trials will clarify which neurosurgical robotic applications can routinely enter clinical practice and can determine the relative extent of the potential clinical benefits granted by the integration and technical refinement of robotic technology.
